# The crucial role of osteocyte-derived RANKL in bone homeostasis

**DOI:** 10.1186/ar3654

**Published:** 2012-02-09

**Authors:** Tomoki Nakashima, Mikihito Hayashi, Hiroshi Takayanagi

**Affiliations:** 1 Department of Cell Signaling, Graduate School of Medical and Dental Sciences, Tokyo Medical and Dental University, Yushima 1-5-45, Tokyo, Japan; 2 Japan Science and Technology Agency (JST), ERATO, Takayanagi Osteonetwork Project, Yushima 1-5-45, Tokyo, Japan

## 

Receptor activator of NF-κB ligand (RANKL; also known as TNFSF11), a TNF family molecule, and its receptor RANK (TNFRSF11A) are key regulators of osteoclast differentiation and function. Aberrant expression of RANKL explains why autoimmune diseases, cancers, leukemia and periodontal disease result in systemic and local bone loss. In particular, RANKL is the pathogenic factor that cause bone and cartilage destruction in arthritis. Inhibition of RANKL function by the natural decoy receptor osteoprotegerin (OPG; also known as TNFRSF11B) or anti-RANKL antibody prevents bone loss in postmenopausal osteoporosis, cancer metastases and arthritis. RANKL also regulates T cell/dendritic cell communications, dendritic cell survival and lymph node organogenesis. Intriguingly, RANKL and RANK play an essential role in the maturation of mammary glands in pregnancy and lactation. Bone homeostasis depends on the coordination of osteoclastic bone resorption and osteoblastic bone formation. We reported that RANKL induces osteoclast differentiation through activating a transcriptional programme mediated by the master transcription factor nuclear factor of activated T cells (NFAT) c1. Although it is well accepted that the RANKL-NFATc1 pathway is crucially important for osteoclast differentiation, little is known about the major cellular source of RANKL in the skeletal tissue. RANKL has been postulated to be mainly expressed by osteoblasts and bone marrow stromal cells. However, here we show that osteocytes embedded within the bone matrix are the critical source of RANKL in bone remodeling. Osteocytes, the most abundant cell type in bone, are thought to orchestrate bone homeostasis by regulating both osteoclastic bone resorption and osteoblastic bone formation, but *in vivo *evidence and the molecular basis for the regulation has not been sufficiently demonstrated. Using a newly established method for the isolation of high-purity dentin matrix protein 1-positive osteocytes from bone, we have found that osteocytes express a much higher amount of RANKL and have a much greater capacity to support osteoclast formation than osteoblasts and bone marrow stromal cells. The crucial role of RANKL expressed by osteocytes was validated by the severe osteopetrotic phenotype observed in mice lacking RANKL specifically in osteocytes. Thus, we provide *in vivo *evidence for the key role of osteocyte-derived RANKL in bone homeostasis, establishing a molecular basis for osteocyte regulation of bone resorption.

